# Executive control of freestyle skiing aerials athletes in different training conditions

**DOI:** 10.3389/fpsyg.2022.968651

**Published:** 2022-09-26

**Authors:** Hui Li, Liancheng Zhang, Jingru Wang, Jie Liu, Yanlin Sun

**Affiliations:** Key Laboratory of Competitive Sport Psychological and Psychological Regulation, Tianjin University of Sport, Tianjin, China

**Keywords:** freestyle skiing aerials, training conditions, executive control, fNIRS, go/nogo task

## Abstract

**Introduction:**

Due to the actual limitation of training conditions, the freestyle skiing aerials winter training term is short. Training tasks such as adaptability training and developing new skills are needed in summer training. When facing different training environments, freestyle skiing aerial athletes’ executive control over their abilities could be affected, which can affect their performance. Therefore, we want to research the effect of training conditions on executive control in freestyle skiing aerials athletes and its neural mechanism.

**Materials and methods:**

Thirty-two freestyle skiing aerials athletes were recruited. We evaluated their executive control and used fNIRS to measure oxygenated hemoglobin concentration changes in the prefrontal cortex during a rapid event-related design go/nogo task with different training condition-activated materials.

**Results:**

Athletes’ behavior control in the summer condition has a lower accuracy than it is in the control condition. Athletes’ behavior control in the summer and winter training conditions had a longer reaction time than that in the control condition. The activation of the bilateral dlPFC and orbitofrontal cortex had a significant main effect across training conditions when freestyle skiing aerial athletes completed executive control tasks. The activation of athletes’ bilateral vlPFC and left dlPFC had an interaction between training conditions and behavioral control.

**Conclusion:**

Different training conditions can lead to freestyle skiing aerial athletes executive control ability to drop, players in different training conditions show less activation on both sides of the vlPFC and orbitofrontal. The bilateral vlPFC and left dlPFC have an integrated effect on behavior inhibition across training conditions.

## Introduction

As a winter Olympic sport, Freestyle skiing aerials are loved by the public for their high flying, flow, beauty and difficulty. Freestyle skiing aerials training is not easy. Athletes need to train on ski slopes, so they must train in locations with snow. The novel coronavirus outbreak has made it difficult for athletes to travel abroad in search of snow. Therefore, athletes choose to train in pools or snow fields, depending on the seasons. They usually choose the pool in summer and choose snow fields in winter. This makes freestyle skiing aerials more economical and convenient. Due to the actual limitation of training conditions, the freestyle skiing aerials winter training term is short. Training tasks such as adaptability training and developing new skills are needed in summer training. It brings not only discomfort in motor skill but also mental problems of adjustment and conversion costs. The summer training ground of freestyle skiing aerials is composed of a slide and pool, which is used to simulate training on snow. However, these two trainings are very different, which can cause problems such as movement technique disturbance and poor integration of summer and winter training.

In the process of completing a movement, the pursuit of “high flying, floating, beautiful and difficult” requires athletes to smooth and stretch their movements to obtain high scores in the competition. Athletes are required to complete a set of movements, including an assist slide, take-off, aerial flip and landing, and are required to land and slide out smoothly at a slope of approximately 38°. Usually, when the athletes do somersaults or turn in the air, it is easy to produce a deviation of spatial orientation perception or decision-making conflict. High-level athletes in snow and ice sports can often make accurate judgments of spatial orientation in the air and adjust their postures and body positions according to their own judgments. In the process of adjusting according to an orientation judgment, athletes are required to react or suppress according to spatial orientation events. This shows the executive control ability of the athletes. Therefore, the difference in executive control ability of freestyle skiing aerials athletes will directly affect their motor skills, thus affecting their performance.

Executive control can be described as the higher order or metacognitive functions that are utilized to regulate a self-directed set of purpose-oriented actions in novel or non-habitual situations (Diamond, 2013). These functions, which also enable an individual to flexibly regulate and control his or her own behavior to achieve goals through the operation of fundamental cognitive processes (Ambrosini et al., 2019), may be regarded as a significant characteristic of elite athletes. Freestyle skiing aerials is a self-paced sports that requires complete actions, including performing glissades, flying, performing somersault, twisting and landing, to be performed. Athletes can correct their postures through their own spatial orientation judgments and deviation movement recognition, which are inseparable from behavioral inhibition. The theory, classification and research paradigm of executive control introduced in a broad sense mostly refer to “cold” executive control, emphasizing the broad cognitive ability of decontextualization. However, what most people need in real life is a general ability to focus on the main contradiction, regulate the current emotion, restrain impulses and control behavior in a targeted and context-adapted way (Diamond, 2013). Zelazo and Müller (2010) introduced emotion and motivation into executive control. They described the cognitive processes required for abstract tasks as “cold” executive control and the regulatory processes under emotional load as “hot” executive control. Freestyle skiing aerial athletes face different training environments, and their performance is influenced by complex sports situations. “Hot” executive control will play a greater role in this process.

One experimental paradigm requiring behavioral inhibition is the go/nogo task, in which participants must press a button in response to a go stimuli and withhold that response to nogo stimuli (Randall and Smith, 2011). Although the go/nogo task is simple in form, it involves stimulus discrimination, response selection, motor preparation, response/inhibition, and conflict/error monitoring. Using go/nogo tasks for vertical spatial orientation may allow effective measurement of behavioral inhibition for freestyle skiing aerial athletes (Goldstein et al., 2007). The participants are asked to respond when stimuli were in one spatial orientation and to inhibit in another spatial orientation. Many studies have found that executive control is closely related to the prefrontal lobe (Stuss, 2011). The brain network involved in executive control is mainly the frontal parietal control network (Niendam et al., 2012), and the main brain regions related to executive control are the dorsolateral prefrontal cortex (dlPFC), inferior frontal gyrus (MFG), anterior cingulate cortex (ACC), ventrolateral prefrontal cortex (vlPFC), parietal lobe, motor cortex and cerebellar cortex (Bellebaum and Daum, 2007). The core components of executive control are related to different regions of the frontal lobe, and different executive control components have task-specific regions. The PFC, vlPFC, dlPFC, pre-SMA and ACC are correlated with the specificity of the inhibitory control task (Fan et al., 2003; Mostofsky and Simmonds, 2008; Smith et al., 2017). Functional near-infrared spectroscopy (fNIRS) is widely used in many fields. During cognitive neural activity, the amount of oxygen carried by cerebral blood flow in relevant brain regions will change. Oxygen is transmitted by hemoglobin in blood, and the concentration of oxygenated hemoglobin (Oxy-Hb) in blood increases in brain activity regions. A decrease in deoxy-Hb concentration. The oxygenated hemoglobin concentration and deaerated hemoglobin concentration affect the scattering of near-infrared light: the sensitivity of oxyhemoglobin is approximately 760 nm and 850 nm for near-infrared light and that of deaerated hemoglobin is different; the former is sensitive to an Oxygenation state, and the latter is more sensitive to an oxygenation state. Therefore, by measuring brain activity in the cortex using scattered light attenuation (optical density, OD), the relative concentrations of oxygenated hemoglobin and deoxygenated hemoglobin in the brain during cognitive activities could be deduced, and the brain regions associated with cognitive activities and their relationships were determined (Irani et al., 2007).

Therefore, fNIRS technology was used in this study to compare the neural activation during go/nogo tasks among freestyle skiing aerialists under different training conditions, to explore the influence of sports conditions on athletes’ executive control and to determine the neural mechanism of executive control.

## Materials and methods

### Participants

Thirty-two athletes aged 12–20 years old (mean age, 15.0 ± 1.8 years) were recruited from two freestyle skiing aerials teams of china, including 16 females and 16 males. All participants had 6.5 ± 3.6 years of sport experience (Table 1). Because the participants belonged to sports teams and lived in groups, they had similar lifestyles and eating habits.

**TABLE 1:** Information of participates.

Sex	Number	Age	Sport experience
Male	16	15.3 ± 2.3	4.8 ± 2.8
Female	16	14.5 ± 0.9	4.2 ± 1.2

### Tasks and procedures

The subjects were required to sit comfortably on an experimental seat in front of a computer (X64 architecture and Windows 10 Professional Workstation System), which was 14 inches, with a visual distance of 100 cm. The experimental program was controlled by Eprime 3.0 running on a Windows 10 Professional Workstation environment. A black cross with a point size of 46 was presented in the center of the blank screen for 500 ms. The stimulus was a skiing icon with a length of 5 cm and a width of 5 cm, which was randomly presented above or below the black cross at 25% of the Y axis for 500 ms. Athletes were asked to respond to the Go stimulus as quickly and accurately as possible and stop the response when the nogo stimulus was presented. If the subject did not respond within 1000 ms, they were regarded as ignoring the response button.

After the introduction, the participants could click any key on the keyboard to enter the experimental exercise, which included 10 go and nogo tasks. After the exercise, the subjects could click “Y” to enter the formal experiment or “N” to repeat the practice until they clearly understood the experimental operation requirements.

**FIGURE 1:**
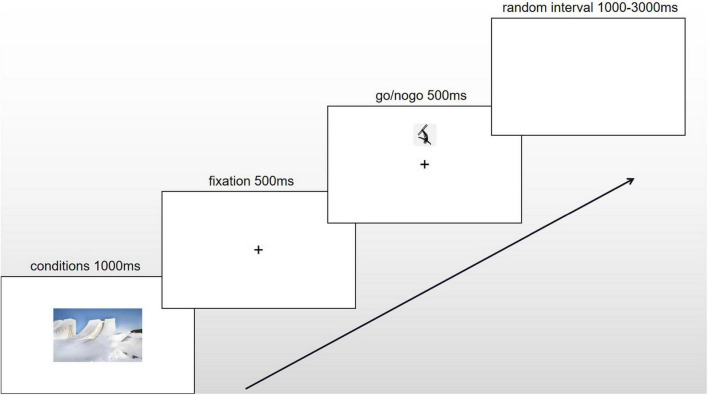
The procedure of one trial under winter conditions.

In the formal experiment, each subject was required to finish 6 blocks, including the up-go/down-nogo experiment under 3 conditions and the down-go/up-nogo experiment under 3 conditions in a random order. It is a within-subject experiment. The up-go/down-nogo and down-go/up-nogo alternated with subjects. Each block of experiments included 60 trials. A condition of activated material was presented 1000ms before each trial, with a random interval of 1000–3000 ms between trials. The activation materials for the winter training condition were 10 photos of the winter skiing venue, and the activation materials for the summer training condition were 10 photos of the summer pool training venue. The blank condition for comparison consisted of a blank screen. Athletes passively observed pictures that simulated their training conditions, which could induce athletes’ recessive emotions under different sports conditions (Goldstein et al., 2007). Stimuli from the same training condition were presented in a single block; interference between stimuli caused by the continuous appearance of different stimuli was avoided by increasing the rest time between blocks (≥ 1 min) and presenting the blocks randomly. Additionally, the athletes were only required to judge the non-emotional attributes (i.e., the position) of the skiing icons when emotion-inducing skiing icons were presented as stimuli, which could also induce athletes’ recessive emotions (Sommer et al., 2008; Yu et al., 2009). The experiment had a rapid event-related design. The ratio of go to nogo stimuli was 1:1 (Figure 1).

### fNIRS recording

**FIGURE 2:**
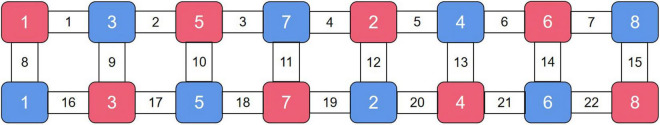
fNIRS setup. We conduct 2 × 8 optode placement for one subject. The red ones are the emitters, and the blue ones are the detectors. The channels between pairs of emitters and detectors are measured.

**FIGURE 3:**
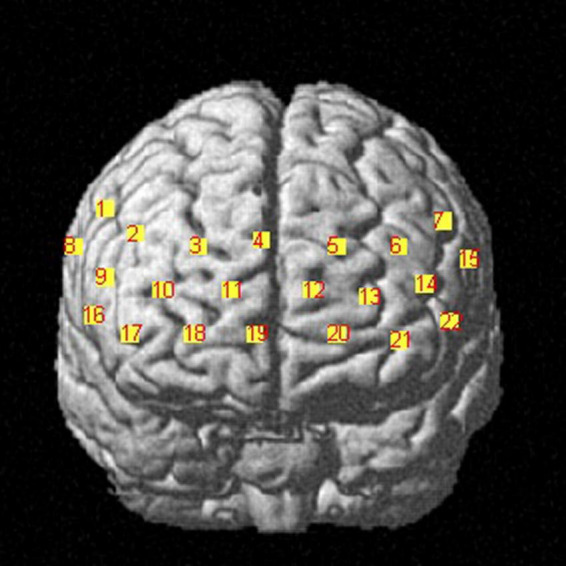
Frontal view of the corresponding anatomical regions of channel configuration. fNIRS data are recorded from the prefrontal cortex, including the right dorsolateral prefrontal cortex, left dorsolateral prefrontal cortex and frontopolar area, localized by the MNI 10-10 system and 3D digitizer.

We used a commercial continuous-wave (CW) fNIRS system (Light NIRS, Shimadzu Corporation, Kyoto, Japan) to measure PFC activity. The absorption values of the three wavelengths (780 nm, 805 nm, and 830 nm) of near-infrared light were measured at a sampling rate of 13.3 Hz and were then transformed into concentration changes in oxyhemoglobin (HbO), deoxyhemoglobin (HbR), and total hemoglobin (HbT) as per the modified Beer–Lambert law (Owen-Reece et al., 1999). Eight emitters and 8 detectors were placed at alternate points on a 2 × 8 grid, enabling us to detect signals from 22 channels (Figure 2). The distance between the two kinds of probes was 3 cm. The center of the probe matrix was placed on Fpz. We applied a three-dimensional digitizer to record the exact spatial coordinates of the four reference points of the 10–10 system (NZ, CZ, AL, RL) as well as the 16 optical probes. We converted these coordinates into locations of the 22 channels in an estimated Montreal Neurological Institute (MNI) space through NIRS-SPM, which calculates the NIRS location maximum likelihood estimate to the Brodman area location (Ye et al., 2009). NIRS-SPM provided a set of probability values for the measured anatomical region of each channel, and a higher probability value represents a more accurate anatomical region. Activity in the right vlPFC was monitored with ch1, ch8, and ch16. Activity in the right dlPFC was monitored with ch2 and ch9. Activity in the frontopolar area was monitored with ch3, ch4, ch5, ch10, ch11, ch12, ch13, ch17, and ch20. Activity in the orbitofrontal area was monitored with ch18, ch19, and ch21. Finally, activity in the left dlPFC was monitored with ch6, ch14, and ch22, while activity in the left vlPFC was monitored with ch7 and ch15 (Figure 3).

### Behavioral data analysis

Behavioral response and behavioral inhibition of freestyle skiing aerials skiers were analyzed by averaging the reaction times of the go task and calculating the accuracy of the go/nogo task. Repeated measures ANOVA was used to assess the effect of training conditions (winter, summer and blank) on the reaction time of the behavioral response. A 3 (training conditions: winter, summer and blank) × 2 (task: go task, nogo task) repeated-measures ANOVA was used to assess the interaction effect of training conditions and behavioral control on the accuracy of the go/nogo task. Training conditions and task were the within-subjects variables. Statistical significance was set at *p* < 0.05. When one of the ANOVA factors was significant, LSD *post hoc* tests were performed.

### fNIRS data analysis

Since HbO signals in the brain have a better signal-to-noise ratio than HbR signals, only HbO data were used for analysis in this study. Based on MATLAB (MathWorks; Natick, MA, United States) Nirs_spm (Statistical Parametric Mapping for Near-infrared Spectroscopy Bio Imaging & Signal Processing lab. KAIST) toolkit for processing the collected HbO data, we used a general linear model (GLM) to analyze the signal. The change in HbO was positively correlated with the degree of brain activation. That is, the greater the estimated parameters of HbO correlation (β) were, the higher the activation degree of the corresponding brain regions of the channel. The wavelet-MDL was used as a high-pass filter to eliminate interference at very low frequencies. The hemodynamic response function (HRF) was used as a low-pass filter. Only correct behavioral responses and activation of behavioral inhibition were analyzed. A 3 (training conditions: winter, summer and blank) × 2 (task: go task, nogo task) repeated-measures ANOVA was used to assess the interaction effect of training conditions and behavioral control on β in the go/nogo task.

## Result

### Behavioral results

**FIGURE 4:**
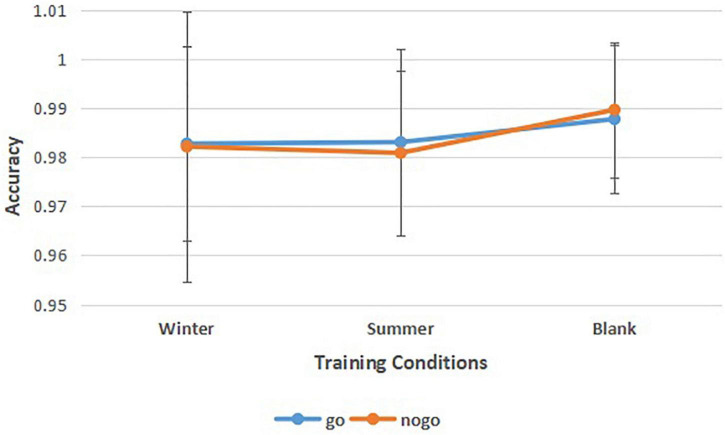
Means of accuracy of go tasks and no-go tasks in three training conditions. Error bars represent the standard deviation.

To find the athletes’ behavior control under different training conditions, we calculated the accuracy of go tasks and nogo tasks in three different training conditions (Figure 4 and Table 2). A 3 (training conditions: winter, summer and blank) × 2 (task: go task, nogo task) repeated-measures ANOVA revealed significant main effects of training conditions [*F* (2,30) = 4.378, *p* = 0.021, $\eta_{p}^{2}$ = 0.226]. The *post hoc* LSD pairwise comparisons indicated that athletes’ behavior control in the summer condition has a lower accuracy than in the control condition (*p* = 0.006). There was no significant interaction effect between behavior control and training condition on accuracy [*F* (2,30) = 0.689, *p* = 0.510, $\eta_{p}^{2}$ = 0.044]. There was no significant main effect of behavior control on accuracy [*F*(1,31) = 0.027, *p* = 0.870, $\eta_{p}^{2}$ = 0.001].

**TABLE 2:** Repeated measures ANOVA of accuracy.

	Winter	Summer	Blank	Repeated-measures ANOVA		
		
	M ± SD	M ± SD	M ± SD	*F*	*p*	$\eta_{p}^{2}$
go	0.9828 ± 0.0197	0.9831 ± 0.0191	0.9878 ± 0.0150			
nogo	0.9822 ± 0.0276	0.9809 ± 0.0167	0.9897 ± 0.0138			
conditions				4.378	0.021	0.226
go/nogo				0.027	0.87	0.001
conditions*go/nogo				0.689	0.51	0.044

**FIGURE 5:**
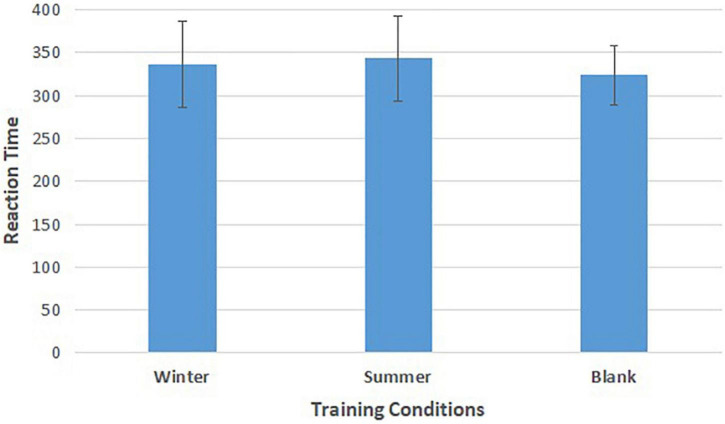
Means of reaction time of go tasks in three training conditions. Error bars represent the standard deviation.

As shown in Figure 5 and Table 3, repeated-measures ANOVA showed significant effects of training conditions (winter, summer and blank) on the reaction time of behavioral response [*F*(2,30) = 4.812, *p* = 0.015, **$\eta_{p}^{2}$** = 0.243]. The *post hoc* LSD pairwise comparisons indicated that the athletes’ behavior control had longer reaction times in the summer condition than in the no condition (*p* = 0.004), and the winter condition had longer reaction times than the control condition (*p* = 0.032).

**TABLE 3:** Repeated measures ANOVA of reaction time.

	Winter	Summer	Blank	Repeated-measures ANOVA		
		
	M ± SD	M ± SD	M ± SD	*F*	*p*	$\eta_{p}^{2}$
go	336.62 ± 50.50	343.32 ± 49.73	323.45 ± 34.80			
conditions				4.812	0.015	0.243

### fNIRS results

**FIGURE 6:**
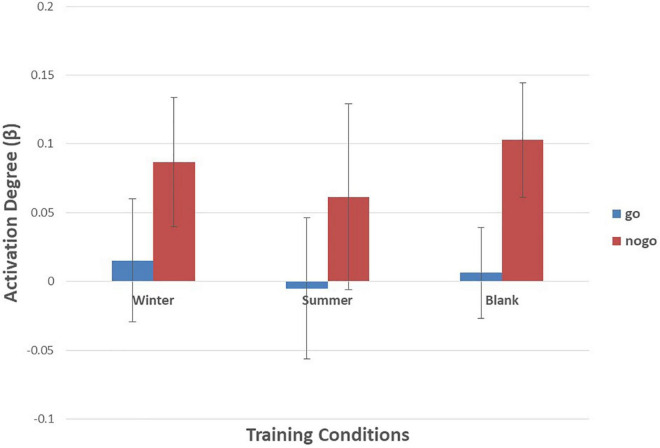
Means of activation degree (β) of ch1 of go tasks and nogo tasks in three training conditions. Error bars represent the standard deviation.

**FIGURE 7:**
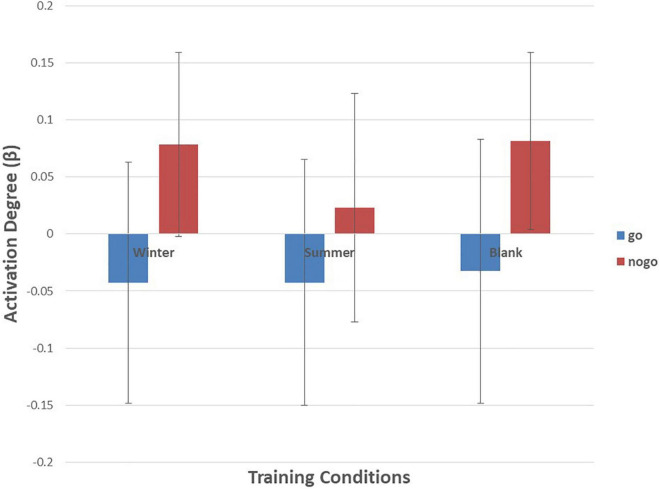
Means of activation degree (β) of ch7 of go tasks and nogo tasks in three training conditions. Error bars represent the standard deviation.

**FIGURE 8:**
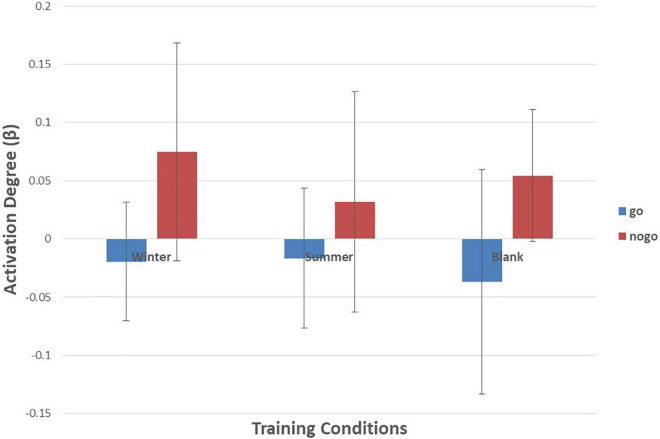
Means of activation degree (β) of ch8 of go tasks and nogo tasks in three training conditions. Error bars represent the standard deviation.

**FIGURE 9:**
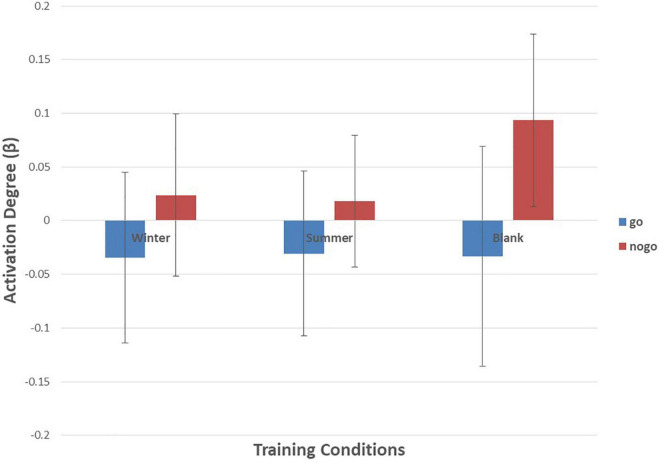
Means of activation degree (β) of ch22 of go tasks and nogo tasks in three training conditions. Error bars represent the standard deviation.

**FIGURE 10:**
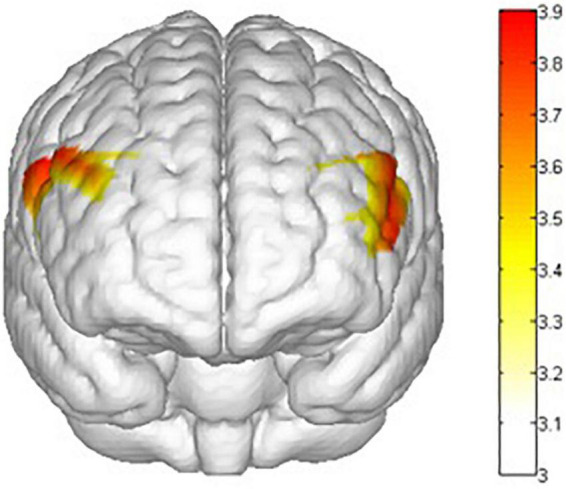
Mapping of interaction effects F value. ch1, ch7, ch8, and ch22 had a lower activation degree (β) in the nogo task in the summer training conditions. All statistical parametric maps were created with a threshold of *p* < 0.05.

As shown in Figures 6–10 and Table 4, a 3 (training conditions: winter, summer and blank) × 2 (task: go task and nogo task) repeated-measures ANOVA on β revealed significant interaction effects in ch1 [*F*(2,30) = 3.989, *p* = 0.029, **$\eta_{p}^{2}$** = 0.210], ch7 [*F*(2,30) = 3.566, *p* = 0.041, **$\eta_{p}^{2}$** = 0.192], ch8 [*F*(2,30) = 4.401, *p* = 0.021, **$\eta_{p}^{2}$** = 0.227] and ch22 [*F*(2,30) = 3.708, *p* = 0.036, **$\eta_{p}^{2}$** = 0.198]. The *post hoc* LSD results revealed a significant effect of training condition in the nogo task on ch1 [*F*(2, 30) = 3.508, *p* = 0.043, **$\eta_{p}^{2}$** = 0.190], ch7 [*F*(2, 30) = 3.308, *p* = 0.050, **$\eta_{p}^{2}$** = 0.181], ch8 [*F*(2, 30) = 4.741, *p* = 0.016, **$\eta_{p}^{2}$** = 0.240], and ch22 [*F*(2, 30) = 14.468, *p* = 0.000, **$\eta_{p}^{2}$** = 0.491]. Simple effect analysis showed that ch1 (*p* = 0.012), ch7 (*p* = 0.014), and ch22 (*p* = 0.000) had a lower activation degree (β) on the nogo task in the summer training condition than in the control condition. The ch8 (*p* = 0.005) had a lower activation degree (β) on the nogo task in the summer training condition than in the winter training condition. Ch22 had a lower activation degree (β) in the nogo task in the winter training condition (*p* = 0.000).

**TABLE 4:** Repeated-measures ANOVA of activation (β).

	Estimated region	Winter	Summer	Blank	Repeated-measures ANOVA		
		
		M ± SD	M ± SD	M ± SD	*F*	*p*	$\eta_{p}^{2}$
go(Ch1)	right vlPFC	0.0153 ± 0.0447	−0.0502 ± 0.0515	0.0064 ± 0.0331			
nogo(Ch1)		0.0867 ± 0.0470	0.0616 ± 0.0675	0.1028 ± 0.0417			
conditions					4.653	0.013	0.131
go/nogo					187.87	< 0.001	0.858
conditions*go/nogo					3.989	0.029	0.21
go(Ch2)	right dlPFC	−0.0133 ± 0.0594	−0.0162 ± 0.0602	−0.0227 ± 0.0630			
nogo(Ch2)		0.0811 ± 0.0477	0.0617 ± 0.0541	0.0952 ± 0.0458			
conditions					5.386	0.01	0.264
go/nogo					66.534	< 0.001	0.682
conditions*go/nogo					3.103	0.06	0.171
go(Ch7)	left vlPFC	−0.0427 ± 0.1058	−0.0426 ± 0.1077	−0.0325 ± 0.1158			
nogo(Ch7)		0.0783 ± 0.0807	0.0234 ± 0.1002	0.0816 ± 0.0778			
conditions					2.32	0.108	0.07
go/nogo					46.31	< 0.001	0.599
conditions*go/nogo					3.566	0.041	0.192
go(Ch21)	orbitofrontal cortex	0.021 ± 0.0938	0.0236 ± 0.0824	0.0568 ± 0.0421			
nogo(Ch21)		0.0179 ± 0.0626	0.0083 ± 0.0556	0.0574 ± 0.0417			
conditions					5.104	0.012	0.254
go/nogo					0.556	0.462	0.018
conditions*go/nogo					0.565	0.574	0.036
go(Ch22)	left dlPFC	−0.0344 ± 0.0796	−0.0308 ± 0.0767	−0.0333 ± 0.1025			
nogo(Ch22)		0.0237 ± 0.0756	0.0178 ± 0.0613	0.0933 ± 0.0802			
conditions					9.242	0.001	0.381
go/nogo					20.59	< 0.001	0.399
conditions*go/nogo					3.708	0.036	0.198

**FIGURE 11:**
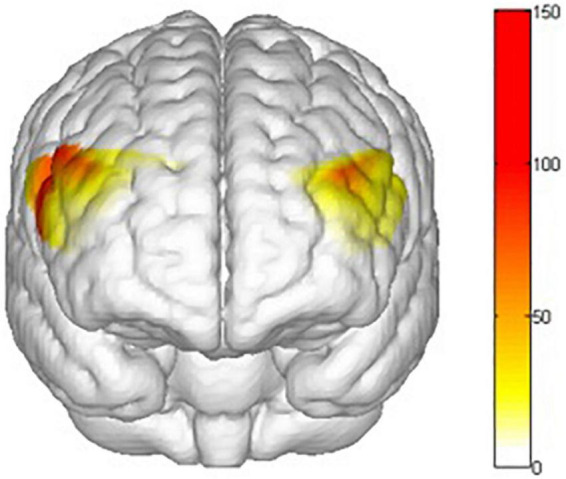
Mapping of the main effects F value of response inhibition activation. The activation degree (β) of the nogo task was higher than that of the go task in the rvPFC, rdPFC, lvPFC, and ldPFC. All statistical parametric maps were created with a threshold of *p* < 0.05.

There are main effects on task (go vs. nogo) in ch1 [*F*(1,31) = 187.870 *p* = 0.000, **$\eta_{p}^{2}$** = 0.858], ch2 [*F*(1,31) = 66.534, *p* = 0.000, **$\eta_{p}^{2}$** = 0.682], ch6 [*F*(1,31) = 73.09, *p* = 0.000, **$\eta_{p}^{2}$** = 0.702], ch7 [*F*(1,31) = 46.310, *p* = 0.000, **$\eta_{p}^{2}$** = 0.599], ch8 [*F*(1,31) = 17.236, *p* = 0.000, **$\eta_{p}^{2}$** = 0.357], ch9 [*F*(1,31) = 38.505, *p* = 0.000, **$\eta_{p}^{2}$** = 0.554], ch14 [*F*(1,31) = 39.660, *p* = 0.000, **$\eta_{p}^{2}$** = 0.561], ch15 [*F*(1,31) = 49.491, *p* = 0.000, **$\eta_{p}^{2}$** = 0.615], ch16 [*F*(1,31) = 86.308, *p* = 0.000, **$\eta_{p}^{2}$** = 0.736] and ch22 [*F*(1,31) = 20.590, *p* = 0.000, **$\eta_{p}^{2}$** = 0.399]. This means that there are behavior inhibition activations in the rvPFC, rdPFC, lvPFC, and ldPFC (Figure 11).

**FIGURE 12:**
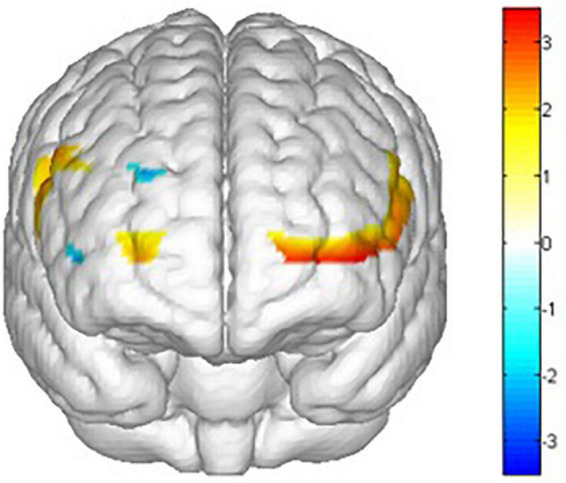
Main effects t value of training conditions activation by comparing control condition and summer conditions (blank > summer). All statistical parametric maps were created with a threshold of *p* < 0.05.

**FIGURE 13:**
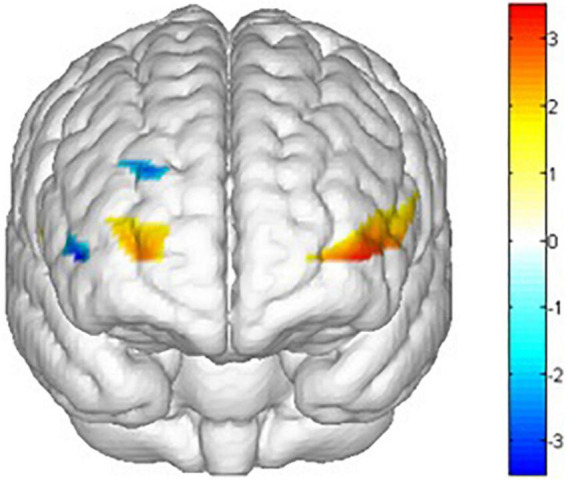
Main effects t value of training conditions activation by comparing control condition and summer conditions (blank > winter). All statistical parametric maps were created with a threshold of *p* < 0.05.

There are main effects on training conditions (winter, summer and blank) in ch1 [*F*(2,62) = 4.653, *p* = 0.013, **$\eta_{p}^{2}$** = 0.131], ch2 [*F*(2,30) = 5.386, *p* = 0.010, **$\eta_{p}^{2}$** = 0.264], ch3 [*F*(2,30) = 3.705, *p* = 0.036, **$\eta_{p}^{2}$** = 0.198], ch8 [*F*(2,30) = 3.999, *p* = 0.029, **$\eta_{p}^{2}$** = 0.210], ch14 [*F*(2,30) = 3.605, *p* = 0.040, **$\eta_{p}^{2}$** = 0.194], ch15 [*F*(2,30) = 3.960, *p* = 0.030, **$\eta_{p}^{2}$** = 0.209], ch16 [*F*(2,30) = 3.541, *p* = 0.042, **$\eta_{p}^{2}$** = 0.191], ch17 [*F*(2,30) = 4.215, *p* = 0.024, **$\eta_{p}^{2}$** = 0.219], ch18 [*F*(2,30) = 3.772, *p* = 0.035, **$\eta_{p}^{2}$** = 0.201], ch20 [*F*(2,30) = 5.118, *p* = 0.012, **$\eta_{p}^{2}$** = 0.254], ch21 [*F*(2,30) = 5.104, *p* = 0.012, **$\eta_{p}^{2}$** = 0.254] and ch22 [*F*(2,30) = 9.242, *p* = 0.001, **$\eta_{p}^{2}$** = 0.381]. The *post hoc* LSD pairwise comparisons indicated that ch1 (*p* = 0.019), ch2 (*p* = 0.03), ch15 (*p* = 0.010), ch16 (*p* = 0.011), ch18 (*p* = 0.029), ch20 (*p* = 0.016), ch21 (*p* = 0.004) and ch22 (*p* = 0.000) had a lower activation degree (β) in the summer training conditions than in the control condition (Figure 12). ch14 (*p* = 0.029), ch16 (*p* = 0.024), ch18 (*p* = 0.009), ch21 (*p* = 0.005) and ch22 (*p* = 0.004) had a lower activation degree (β) in the winter training conditions than in the blank condition (Figure 13). The activation degree (β) in the winter training conditions was higher than that in the summer training conditions in ch1 (*p* = 0.023), ch2 (*p* = 0.019), ch8 (*p* = 0.019), and ch20 (*p* = 0.011). ch3 (*p* = 0.010) and ch17 (*p* = 0.007) had a lower activation degree (β) in the control condition than in winter. ch3 (*p* = 0.024) and ch17 (*p* = 0.032) had a lower activation degree (β) in the control condition than in summer. Only ch14 had a lower activation degree (β) in winter training conditions than in summer.

## Discussion

### Executive control and neural activation of freestyle skiing aerials athletes in different training conditions

The behavioral results showed a significant main effect of executive control accuracy in different training conditions, and the executive control accuracy in the summer training conditions was significantly lower than that in the no training conditions. The behavioral response times varied significantly across training conditions, and the behavioral response time in summer training conditions and winter training conditions was significantly longer than that in non-training conditions. This result indicated that training conditions impact executive control during freestyle skiing aerials. Both summer training conditions and winter training conditions reduce the executive control performance of athletes. Athletes’ performance control accuracy does not exhibit an interaction effect on training conditions and behavior control. The impact of training conditions is embodied in two aspects: behavioral responses and behavioral inhibition. Athletes can neither respond to the go task quickly nor inhibit the nogo task accurately. The decline in executive control ability leads to lower self-control for freestyle skiing aerials athletes in actual training, meaning they cannot accurately control their movements and make correct and effective behavioral responses.

The fNIRS results showed that the activation of the bilateral dlPFC and orbitofrontal cortex had a significant main effect across training conditions when freestyle skiing aerials athletes completed executive control tasks. Regarding the role of the orbitofrontal cortex in executive control in different training conditions, it can be speculated that it is related to previous findings that the amygdala transmits the information of interaction between emotion and behavioral control to the orbitofrontal cortex, and the orbitofrontal cortex processes and codes this information before making the decision of behavioral response or inhibition (Yang et al., 2010). In this experiment, the orbitofrontal cortex was not found to participate in the interaction between the training conditions and the behavior control but showed different activation degrees in the different training conditions, and the activation degree in the training conditions was great than in the non-training conditions. We think that the orbitofrontal cortex and amygdala are directly connected and indirectly connected through the dorsomedial thalamus (Roberts and Wallis, 2000). The activation of emotion control by the amygdala occupies resources of the orbitofrontal cortex, resulting in decreased activation of the orbitofrontal cortex. In view of this, the role of the orbitofrontal cortex and amygdala in executive control under different training conditions needs to be further studied.

### Lateral prefrontal activation of freestyle skiing aerial athletes during behavioral inhibition

The results showed that the bilateral dlPFC and bilateral vlPFC were significantly activated in athletes during behavioral inhibition, which was consistent with the results of previous studies. Previous NIRS analysis studies on go/nogo tasks generally used block design to compare nogo blocks with go blocks (Herrmann et al., 2005; Kaga et al., 2020; Wu et al., 2021). In the nogo block, half of the stimuli are go responses, the other half are nogo responses, and all of the stimuli require response in the go block. The block design could not well distinguish neural activation in the go task from that in the nogo task and could not exclude trials with incorrect responses from the experimental data. To study the specificity of behavioral inhibition relative to behavioral response, we used a rapid event-related design presented by random stimulus, which can improve the accuracy of brain functional localization of behavioral inhibition. The results showed that the dlPFC was closely related to behavioral inhibition, and the role of the vlPFC in behavioral inhibition was also found.

### Integration of the vlPFC and dlPFC on behavioral inhibition across training conditions

Repeated measures ANOVA of the fNIRS results showed that the activation of athletes’ bilateral vlPFC had an interaction between training conditions and behavioral control. In summer training conditions, the activation of the bilateral vlPFC was significantly reduced in the athletes during behavioral inhibition. This suggests that the bilateral vlPFC of freestyle skiing aerials athletes plays an integrated role in behavioral inhibition under different training conditions. Only one channel in the left dlPFC showed an integration effect of different training conditions on behavioral inhibition. Previous researchers have used emotional go/nogo tasks to find that behavioral inhibition activates the dlPFC, so it is believed that the integration of emotion and cognition may occur in the dlPFC, which is inconsistent with the results of this study. Does it mean that the influence mechanism of different training conditions on athletes’ behavioral inhibition has nothing to do with emotion? In previous studies using the emotional go/nogo task, the emotional stimulus and the target stimulus of the behavioral inhibition task were the same. This led to confusion of the emotional cue with the inhibition task, which did not allow for separation of the emotional processing from the integrated processing of emotion and behavioral inhibition (Sakatani et al., 2006; Hare et al., 2008). In this study, the emotional interference method was used to create the sport conditions, and at the same time, the emotional stimulus was separated from the target stimulus in the behavior inhibition task. Meanwhile, stimuli with the same potency were kept in a single block, and the interference between stimuli caused by the continuous appearance of different stimuli titers was avoided by appropriately increasing the rest time between blocks. The results more objectively showed that the vlPFC was involved in the integration of training conditions and behavioral inhibition in athletes. However, it is still uncertain whether the vlPFC was an important area of the integration of emotion and cognition, and the same experimental paradigm should be used for a comparative study in the future. So we could not make sure that the influence mechanism of executive control of freestyle skiing aerial athletes under different training conditions is related to emotion.

## Conclusion

The fNIRS technology was used in this study to compare the neural activation during go/nogo tasks among freestyle skiing aerialists under different training conditions, to explore the influence of sports conditions on athletes’ executive control and to determine the neural mechanism of executive control. The following conclusions were drawn: different training conditions can lead to a drop in the executive control ability of freestyle skiing aerials athletes, and athletes show less activation on both sides of the vlPFC and orbitofrontal under different training conditions. The bilateral vlPFC and bilateral dlPFC were involved in the athletes’ behavioral inhibition, and the activation degree was improved. The bilateral vlPFC and left dlPFC have an integrated effect on behavior inhibition in different training conditions.

## Data availability statement

The raw data supporting the conclusions of this article will be made available by the authors, without undue reservation.

## Ethics statement

The studies involving human participants were reviewed and approved by Tianjin University of Sport Research Ethics Committee. Written informed consent from the participants’ legal guardian/next of kin was not required to participate in this study in accordance with the national legislation and the institutional requirements.

## Author contributions

HL, LZ, and YS contributed to conception and design of the study. JL conducted the experiment. JW organized the database. HL performed the statistical analysis and wrote the manuscript. All authors contributed to manuscript revision, read, and approved the submitted version.
